# Draft Genomes of Geographically Distinct Strains and Progeny of the Ectomycorrhizal Basidiomycete *Laccaria bicolor*

**DOI:** 10.7150/jgen.130158

**Published:** 2026-01-30

**Authors:** Francis M. Martin, Emmanuelle Morin, Alan Kuo, Igor Miquel, Jessy Labbé, François Le Tacon, Laure Fauchery, Annegret Kohler, William Andreopoulos, Alex Copeland, Hui Sun, Asaf Salamov, Anna Lipzen, James Han, Kurt LaButti, Andrew Tritt, Kerry Barry, Igor V. Grigoriev

**Affiliations:** 1 Université de Lorraine, INRAE, UMR Interactions Arbres/Microorganismes, INRAE-Grand Est-Nancy, 54280 Champenoux, France.; 2 U.S. Department of Energy Joint Genome Institute, Lawrence Berkeley National Laboratory, Berkeley, CA 94720, USA.; 3 Biosciences Division, Oak Ridge National Laboratory, 1 Bethel Valley Road, Oak Ridge, TN 37831, USA.; 4 Department of Plant and Microbial Biology, University of California Berkeley, Berkeley, CA 94720, USA.

**Keywords:** Ectomycorrhizal fungi, *Laccaria bicolor*, pangenome, comparative genomics, genetic diversity, symbiosis

## Abstract

The ectomycorrhizal fungus *Laccaria bicolor* is a key symbiotic mutualist in forest ecosystems, where it enhances nutrient uptake and promotes the growth of host trees. Here, we present genome assemblies of 14 geographically distinct strains and progeny of *L. bicolor*, providing new insights into the intraspecific genomic diversity. Pangenome analysis revealed substantial variation in assembly size (42-96 Mbp), gene content (16,084-26,800 genes), and single nucleotide polymorphism (SNP) density (0.04-12.08 SNPs/kb). This variation likely reflects genuine biological differences among strains adapted to diverse environmental conditions, although differences in assembly quality and repeat content may also play a role. These genomic resources, comprising draft genome assemblies with comprehensive annotations, will facilitate comparative studies of the genetic diversity and functional traits underlying the ecological success of this model ectomycorrhizal fungus.

## Introduction

Ectomycorrhizal fungi form mutualistic associations with most tree species in forest ecosystems and play essential roles in nutrient cycling and tree growth 1. *Laccaria bicolor*, a member of the Hydnangiaceae family within the order Agaricales (Basidiomycota), has long served as an experimental model in mycorrhizal research 2. The genome of* L. bicolor* strain S238N-H82 comprises a 60.7-megabase assembly containing approximately 23,130 predicted protein-coding genes 3. Analysis of this genome and associated transcriptomes has revealed gene networks involved in symbiosis development, including effector-type secreted proteins known as mycorrhiza-induced small secreted proteins (MiSSPs), which play critical roles in symbiotic establishment 4,5.

Notably, the *L. bicolor* genome encodes a reduced number of carbohydrate-active enzymes (CAZymes) involved in plant cell wall degradation while retaining the ability to degrade microbial cell wall polysaccharides. This pattern reflects the dual saprotrophic and biotrophic lifestyles characteristic of ectomycorrhizal fungi 3, and the loss of genes encoding plant cell wall-degrading enzymes has been confirmed across dozens of ectomycorrhizal species 6.

Understanding intraspecific variability in genomic features is essential for comprehensive comparative and evolutionary genomic analyses of ectomycorrhizal fungi 7. Analysis of the *L. bicolor* pangenome and its intraspecific genetic variability can provide crucial insights into the genomic diversity, adaptation, and evolution of this ecologically important, symbiotic fungus. Our pangenomic analysis identified core genes shared among geographically distinct strains and genes shared among the progeny of the *L. bicolor* S238N lineage. We also characterised accessory genes that may facilitate local adaptation to diverse environments.

## Materials and Methods

### Fungal Strains and Culture Conditions

Strains D101 and S238O of *L. bicolor* were collected in Quebec Province, Canada (no known host), whereas strain DR170 was collected in the Upper Peninsula of Michigan, USA, beneath *Pinus resinosa*. Strain S238N originated from a basidiocarp collected beneath *Tsuga mertensiana* at Crater Lake, Oregon, USA, and subsequently produced basidiocarps with Douglas fir seedlings in the INRAE-Nancy greenhouse. Strains 81306, Cham3, and N203 were collected from Douglas fir plantations at Barbaroux, Saint-Brisson, and Chammet in the Auvergne region.

The S238N-H82, S238N-H70, and S238N-H53 strains are sib-monokaryons derived from the parental strain S238N. The S238N-H82 × H70 strain was generated by crossing the homokaryotic strains S238N-H70 and S238N-H82. Strain S238N 93.12 originated from* L. bicolor* S238N after 20 years of storage in liquid N2.* L. bicolor* CBS 594.89 (origin unknown) and CBS 559.96 (collected under *Pinus sylvestris* in the Netherlands) were obtained from the CBS fungal collection (Westerdijk Institute, Utrecht, NL).

Free-living vegetative mycelium of each* L. bicolor* strain was grown for approximately three weeks in Pachlewski medium 8 at 25°C on agar. Mycelial colony edges were sampled, snap-frozen in liquid N_2_, and ground into a fine powder.

### DNA Extraction, Genome Sequencing, and Assembly

Total genomic DNA was extracted from three grams of vegetative mycelium using a CTAB-based protocol 3 and purified using Qiagen genomic-tip 500/G columns, following the manufacturer's instructions 3. All new *L. bicolor* genomes and transcriptomes were sequenced using Illumina technology, with some genomes additionally combined with PacBio sequencing technology.

For transcriptomes, stranded cDNA libraries were generated using the Illumina TruSeq Stranded RNA LT Kit. mRNA was purified from one µg of total RNA using magnetic beads containing poly T oligonucleotides, fragmented, and reverse-transcribed using random hexamers and SSII (Invitrogen), followed by second-strand synthesis. Fragmented cDNA was treated with end-repair, A-tailing, adapter ligation, and eight cycles of PCR amplification.

For all genomes, Illumina regular fragment libraries were produced from 100 ng of DNA sheared to 300 bp using a Covaris LE220 (Covaris) and size-selected using SPRI beads (Beckman Coulter). Fragments were treated with end-repair, A-tailing, and ligation of Illumina-compatible adapters (IDT, Inc.) using the KAPA-Illumina library creation kit (KAPA Biosystems).

Additionally, for* L. bicolor* 81306, D101, DR170, N203, S238O, S238N, S238N-H82, S238N-H70, and S238N-H53, Illumina 4 kb Long-Mate Pair (LMP) CLRS libraries were produced from 5 µg of DNA sheared using a Covaris g-TUBE (Covaris) and gel size-selected for 4 kb. The sheared DNA was treated with end repair and ligated to biotinylated adapters containing *loxP*. Adapter-ligated DNA fragments were circularised via recombination using a *Cre* excision reaction (NEB). Circularised DNA templates were randomly sheared using the Covaris LE220 (Covaris) and treated with end repair and A-tailing using the KAPA-Illumina library creation kit (KAPA Biosystems), followed by immobilisation of mate pair fragments on streptavidin beads (Invitrogen). Illumina-compatible adapters (IDT, Inc.) were ligated to the mate pair fragments, and 10-12 cycles of PCR were used to enrich the final library (KAPA Biosystems).

Both genome and transcriptome libraries were quantified using KAPA Biosystem's next-generation sequencing library qPCR kit (Roche) and run on a Roche LightCycler 480 real-time PCR instrument. Quantified libraries were multiplexed, and pooled libraries were prepared for sequencing on the Illumina HiSeq platform using a TruSeq paired-end cluster kit, v3 or v4, with Illumina's cBot instrument to generate the clustered flow cells. Sequencing was performed on Illumina HiSeq 2000 or 2500 sequencers using HiSeq TruSeq SBS sequencing kits, v3 or v4, following a 2×150 bp (2×100 bp for LMP) indexed run recipe.

RNA-Seq reads were filtered and trimmed for contamination and quality assessment. Using BBDuk (https://sourceforge.net/projects/bbmap/), raw reads were evaluated for artefact sequences by k-mer matching (k-mer = 25), allowing one mismatch, and the detected artefacts were trimmed from the 3' end. RNA spike-in reads, PhiX reads, and reads containing Ns were also removed. Quality trimming was performed using the Phred trimming method, which was set at Q6. Following trimming, reads below the length threshold were removed (minimum length 25 bases or one-third of the original read length, whichever was longer). Filtered reads were assembled into consensus sequences using Trinity ver. 2.1.1.

All DNA reads filtered for artefact/process contamination were assembled with AllPathsLG versions R41043, R44849, R47710, and R49403 9. For CBS 594.89, CBS 559.96, S238N-H82×H70, and Cham3, which lacked LMP data, Velvet 10 assemblies were generated and used to produce *in silico* LMP libraries with inserts of 3000 ± 300 bp size.

The genomes of strains D101, DR170, N203, S238N 93.12, S238N-H82, and S238N-H70 were also improved using PacBio sequencing. Unamplified libraries were generated using the Pacific Biosciences standard template preparation protocol for creating >10 kb libraries (>20 kb for S238N 93.12 and 3 kb for DR170). Five µg of gDNA was used for each library preparation. DNA was sheared using Covaris g-TUBEs to generate fragments >10 kb (3 kb for DR170). Sheared DNA fragments were prepared using the Pacific Biosciences SMRTbell Template Preparation Kit. The fragments were treated with DNA damage repair, the ends were repaired to be blunt-ended and 5' phosphorylated, and Pacific Biosciences hairpin adapters were ligated to create the SMRTbell templates for sequencing. Templates were purified using exonuclease treatments and size-selected using AMPure PB beads or the Sage Sciences Blue Pippin Size-Selection system for S238N 93.12. PacBio sequencing primers were annealed to the SMRTbell template library, and Version P4 or P6 sequencing polymerase was bound. The prepared SMRTbell template libraries were sequenced on a Pacific Biosciences RSII sequencer using Version C2 or C4 chemistry and 1×120 or 1×240 sequencing movie runtime.

AllPathsLG assemblies were patched using error-corrected PacBio data with PBJelly v12.9.14 11 and polished with Quiver version smrtanalysis_2.3.0.140936. p5 (https://github.com/PacificBiosciences/GenomicConsensus/). For S238N 93.12, PacBio data were filtered with smrtanalysis and assembled with Falcon v.20150310 (https://github.com/PacificBiosciences/FALCON), and improved with finisherSC v. 1.0 12 and polished with Quiver.

### Gene Prediction and Annotation

Gene models were predicted using the JGI Annotation Pipeline, which integrates multiple gene prediction algorithms with RNA-seq evidence 13. Functional annotation was performed by comparing the predicted proteins with the Pfam, KOG, and KEGG databases. Secreted proteins were predicted in the genomes of *L. bicolor* strains using a custom bioinformatic pipeline 15.

### Orthology and Pangenome Analysis

Orthology of the protein repertoires was assessed using FastOrtho/Silix (50% identity, 50% coverage) to identify core genes (present in all strains), dispensable genes (present in at least two strains), and strain-specific genes. Strain-specific genes were further compared with all fungal genomes at JGI to identify species-specific genes.

### Phylogenomic Analysis

Phylogenomic analysis was performed as described by 3. Briefly, we identified clusters containing single-copy genes, aligned each cluster with MAFFT, eliminated ambiguous regions (containing gaps and poorly aligned regions), and concatenated the single-gene alignments with GBLOCKS. We constructed a phylogenetic tree using RAxML with the standard algorithm, the PROTGAMMAWAG model of sequence evolution, and 1000 bootstrap replicates. The final species tree was estimated using ASTRAL 14, with 2,038 unrooted gene trees.

### SNP Prediction

Single nucleotide polymorphisms (SNPs) were predicted for 16 strains relative to the reference genome (*Laccaria bicolor* v2.0) using BWA-MEM for read mapping and SAMtools/BCFtools for variant calling, as described previously 7. The repeated regions were not masked. A phylogenetic tree was generated using RAxML 7.7.2 with the GTRGAMMA model and 500 bootstrap replicates.

## Results

### Genome Assembly and Annotation Statistics

The genome assembly sizes of *L. bicolor* strains ranged from 42.12 to 96.43 Mbp (Table 1). The number of predicted genes ranged from 16,084 to 26,800, with average protein lengths between 310 and 397 amino acids across all strains. Pfam domains were identified in 6,192-14,251 genes per strain (Table 2). This considerable variation in assembly size and gene number likely reflects the differences in assembly quality, repeat content, and genuine biological variation among strains.

### Pangenome Analysis and Gene Orthology

**Figure 1:**
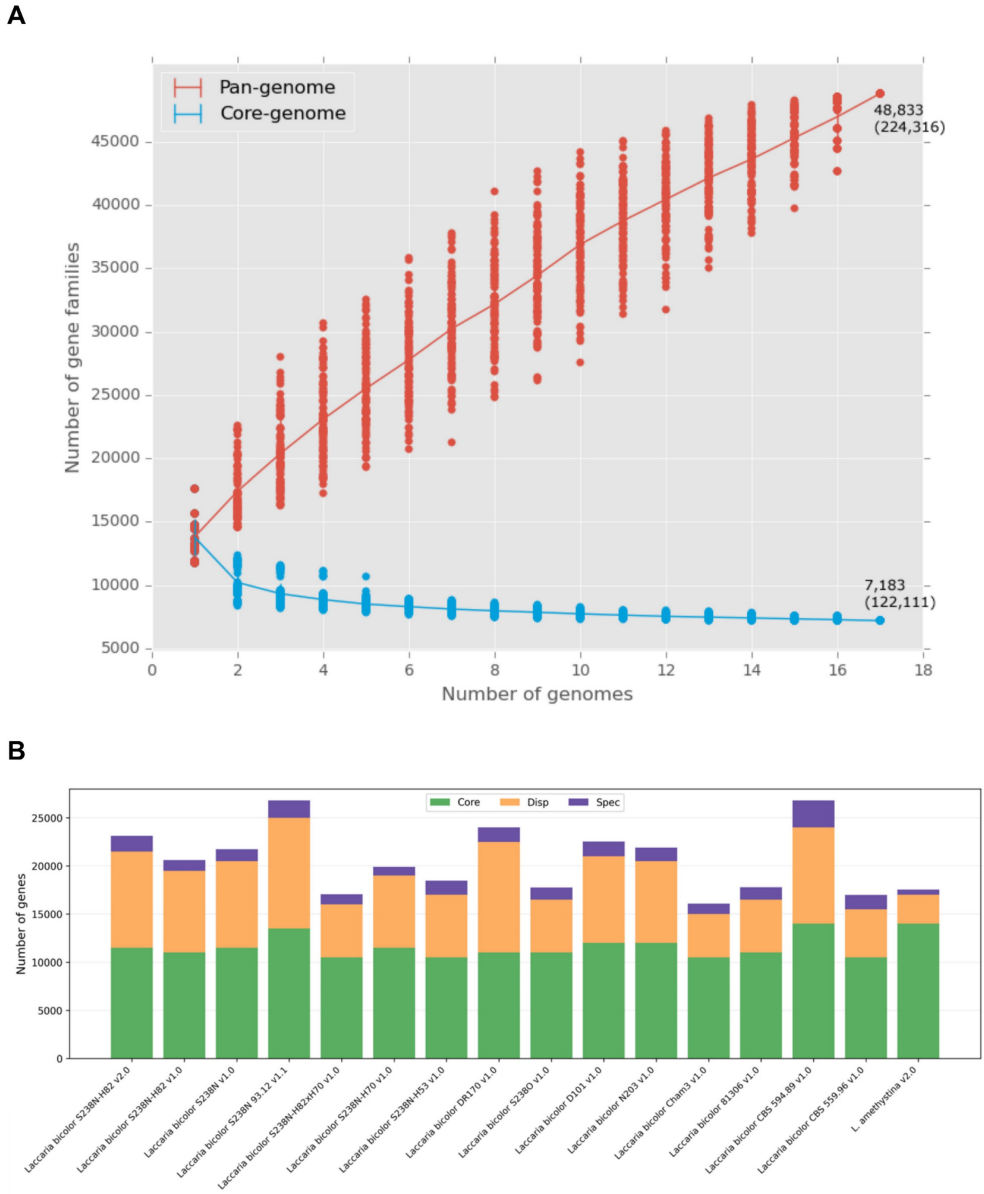
** Core and pangenomes of *Laccaria bicolor*. (A)** Accumulation curves showing how the pangenome size (red) increases and the core genome size (blue) decreases with each additional genome. **(B)** Distribution of core (green), dispensable (orange), and strain-specific (purple) genes across the strains. Stacked bar chart representation.

Pangenome analysis revealed substantial genetic diversity among the 14* L. bicolor* strains (Figure 1A). The core genes (present in all strains) ranged from 10,516 to 14,568 per strain, the dispensable genes ranged from 2,937 to 10,361, and the strain-specific genes ranged from 228 to 3,708 (Figure 1B). When including the related species *L. amethystina*, the pangenome encompassed 48,833 gene families, whereas the core genome stabilised at approximately 7,183 gene families. The substantial number of dispensable and strain-specific genes highlights the functional diversity within *L. bicolor*, potentially enabling adaptation to different soil and climatic conditions.

### Phylogenomic Relationships

**Figure 2:**
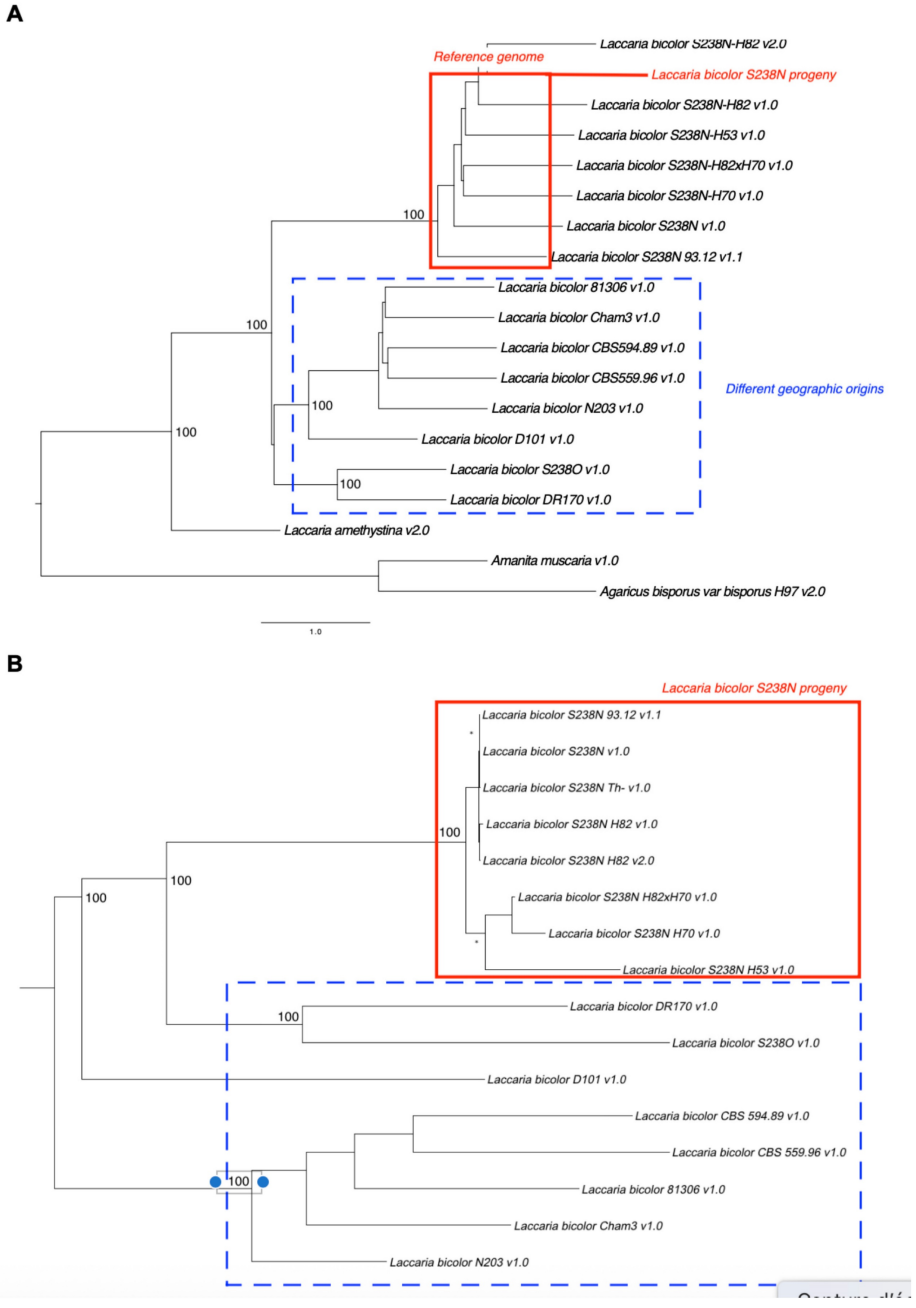
**(A)** Phylogenomic tree of *Laccaria* isolates based on 2,038 single-copy orthologous gene trees inferred using ASTRAL. The tree included 16 *L. bicolor* strains, one *L. amethystina* strain, and two outgroup species (*Agaricus bisporus* and *Amanita muscaria*). Branch support values are shown at the major nodes. **(B)** Maximum likelihood phylogeny of *L. bicolor* strains based on genome-wide SNP data, constructed using RAxML with the GTRGAMMA model and 500 bootstrap replicates.

Phylogenomic analysis based on 2,038 single-copy orthologues clearly distinguished *L. bicolor* strains from *L. amethystina*, as well as from the more distantly related Agaricales, *Agaricus bisporus* and *Amanita muscaria* (Figure 2A). Several distinct clades were evident within* L. bicolor*. The parental strain S238N and its progeny (homokaryotic and dikaryotic strains) formed a well-supported, monophyletic group. Other distinct clusters indicate the geographic structuring of genetic diversity within species.

### Single Nucleotide Polymorphism Analysis

SNP analysis revealed substantial variations among the strains (Figure 2B). SNP density ranged from 3.15 SNPs/kb (S238N-H82 v.1.0 vs. S238N-H82 v2.0) to 12.08 SNPs/kb (DR170 vs. S238N-H82 v2.0). The proportion of SNPs located within the coding regions varied between 44.57% and 55.34%. The SNP-based phylogeny closely corresponded to the phylogenomic tree derived from conserved proteins (Figure 2A).

The substantial sequence divergence (>1%) observed in certain strains, such as *L. bicolor* DR170, likely reflects geographic isolation and the structuring of genetic diversity within the species. Such levels of divergence raise questions about whether some strains warrant recognition as distinct taxonomic entities.

### Secretome Characteristics

All strains possessed substantial complements of genes encoding secreted carbohydrate-active enzymes (CAZymes; 111-278 genes), lipases (8-17), proteases (40-97), and small secreted proteins (371-606). Complete annotations are available at the *Laccaria* pangenome portal of MycoCosm. Variations in the number of secreted proteins may reflect strain-specific adaptations to different hosts and environmental conditions.

## Discussion

This study presents a comprehensive pangenomic analysis of *Laccaria bicolor*, substantially expanding the genomic resources available for this model ectomycorrhizal species. Our analysis revealed that *L. bicolor* possesses a large accessory genome, with approximately 7,183 core gene families and up to 48,833 total gene families. This open pangenome structure is consistent with species inhabiting diverse environments and associated with multiple host species.

The substantial variation in genome size (42-96 Mbp) and gene content (16,084-26,800 genes) among strains suggests genuine biological differences, although assembly quality may contribute to some of this variation. Strain CBS 594.89, with the largest genome (69.98 Mbp) and highest gene count (26,800), may represent a strain with expanded gene families and elevated transposable element activities.

SNP analysis revealed varying levels of divergence among strains, indicating that our collection captured both recent derivatives of common laboratory strains and geographically isolated populations. The variation in small secreted protein (SSP) repertoires (371-606 genes) was particularly noteworthy. Given the importance of specific MiSSPs in symbiotic establishment 4,5,16,17, strain-specific SSP repertoires may reflect adaptations to different host species or environmental conditions. A detailed functional enrichment analysis of core, accessory, and strain-specific genes, including CAZyme and MiSSP distribution across the pangenome, will be addressed in future studies.

These genomic resources will enable future studies on comparative effector biology, local adaptation, population genomics, functional validation of symbiosis-related genes, and synthetic community. This study contributes to the growing body of fungal pangenome studies and demonstrates the value of population-scale genomics in understanding the ecology and evolution of mycorrhizal fungi. The substantial genetic diversity within* L. bicolor* suggests considerable potential for adaptation to changing environmental conditions, with implications for conservation strategies and biotechnological applications, including reforestation efforts and optimisation of plant-fungal partnerships for sustainable forestry.

## Figures and Tables

**Table 1:** Summary statistics of genome assemblies

Strain / assembly version	Assembly (Mbp)	Contigs #	N50	L50 (Kbp)	Scaffolds #	N50 (#)	L50 (Kbp)	Repeat (%)	GC (%)
*Laccaria bicolor* S238N-H82 v2.0	60.71	584	50	342.8	55	5	3262.84	24.80	46.95
*Laccaria bicolor* S238N-H82 v1.0	64.88	4399	180	71.0	665	21	786.28	21.20	46.97
*Laccaria bicolor* S238N v1.0	67.66	6556	636	21.3	1577	113	160.33	21.68	47.27
*Laccaria bicolor* S238N 93.12 v1.1 *	96.43	1034	109	236.9	1034	109	236.92	22.86	46.84
*Laccaria bicolor* S238N-H82xH70 v1.0	42.12	4436	404	24.1	3788	332	28.50	14.13	47.90
*Laccaria bicolor* S238N-H70 v1.0	57.05	2269	133	107.9	1564	96	150.93	22.86	47.34
*Laccaria bicolor* S238N-H53 v1.0	51.78	3014	219	57.3	1284	88	157.00	20.70	47.49
*Laccaria bicolor* DR170 v1.0	79.07	7156	756	29.1	1941	185	107.45	25.97	47.00
*Laccaria bicolor* S238O v1.0	57.06	3633	214	56.1	1370	68	195.42	22.20	47.31
*Laccaria bicolor* D101 v1.0	70.03	4003	257	62.0	2920	171	106.84	22.37	47.31
*Laccaria bicolor* N203 v1.0	69.63	2759	184	80.3	1866	109	143.29	25.25	47.03
*Laccaria bicolor* Cham3 v1.0	44.65	3687	353	30.2	3327	338	32.50	15.67	47.71
*Laccaria bicolor* 81306 v1.0	50.95	2780	209	57.7	946	74	194.83	18.47	47.51
*Laccaria bicolor* CBS 594.89 v1.0	69.98	4627	309	50.6	4530	299	52.42	8.59	48.44
*Laccaria bicolor* CBS 559.96 v1.0	43.46	3844	373	27.4	3721	364	28.44	15.28	47.71
*Laccaria amethystina* LaAM-08-1 v2.0	52.58	1999	151	89.3	1299	112	121.59	21.88	46.62
*Agaricus bisporus* var *bisporus* H97 v2.0	30.20	254	35	262.5	29	6	2334.61	2.19	46.48
*Amanita muscaria* Koide v1.0	40.70	3814	266	30.0	1101	54	145.60	6.08	47.55

* Sequencing and assembly of the dikaryotic strain

**Table 2:** Summary statistics for the annotated genomes

Strain	# of genes	avg. exon length (nt)	avg. intron length (nt)	Exons per gene	avg. protein length (aa)	# genes w/ Pfam domain	BUSCO score*
*Laccaria bicolor* S238N-H82 v2.0	23132	220	92	5.28	356	6920	96.5
*Laccaria bicolor* S238N-H82 v1.0	20614	210	93	5.4	367	NA	96.4
*Laccaria bicolor* S238N v1.0	21724	227	80	5.04	351	6876	95.9
*Laccaria bicolor* S238N-H82xH70 v1.0	17045	230	75	5.28	372	6192	96.7
*Laccaria bicolor* S238N-H70 v1.0	19903	224	83	5.31	365	6681	95.1
*Laccaria bicolor* S238N-H53 v1.0	18468	230	76	5.29	373	6437	96.8
*Laccaria bicolor* DR170 v1.0	24004	198	78	5.33	310	8193	85.7
*Laccaria bicolor* S238O v1.0	17767	220	79	5.29	388	6514	95.8
*Laccaria bicolor* D101 v1.0	22538	237	86	5.02	360	7177	92.1
*Laccaria bicolor* N203 v1.0	21909	213	86	5.33	343	6720	94.1
*Laccaria bicolor* Cham3 v1.0	16084	217	74	5.32	386	6192	96.2
*Laccaria bicolor* 81306 v1.0	17791	234	81	5.34	378	6413	99.7
*Laccaria bicolor* CBS 594.89 v1.0	26800	224	70	5.70	397	14251	94.3
*Laccaria bicolor* CBS 559.96 v1.0	16977	236	69	5.28	371	7631	97.0
*Laccaria amethystina* LaAM-08-1 v2.0	17553	242	70	5.12	364	6174	97.7
*Agaricus bisporus* var *bisporus* H97 v2.0	10432	232	72	6.05	426	5359	98.0
*Amanita muscaria* Koide v1.0	18153	253	73	4.54	328	6244	97.0

* BUSCO Agaricomycetes gene set
